# Attitudes and perceptions towards novel objective measures of ARV-based vaginal ring use: Results from a global stakeholder survey

**DOI:** 10.1371/journal.pone.0180963

**Published:** 2017-07-14

**Authors:** Randy M. Stalter, Jenae Tharaldson, Derek H. Owen, Eunice Okumu, Thomas Moench, Natasha Mack, Elizabeth E. Tolley, Kathleen M. MacQueen

**Affiliations:** 1 Contraceptive Technology Innovation Department, FHI 360, Durham, North Carolina, United States of America; 2 Global Health Research Department, FHI 360, Durham, North Carolina, United States of America; 3 ReProtect, Inc., Baltimore, Maryland, United States of America; Yale University Yale School of Public Health, UNITED STATES

## Abstract

Results of recent microbicide and pre-exposure prophylaxis clinical trials have shown adherence to be a significant challenge with new HIV prevention technologies. As the vaginal ring containing dapivirine moves into two open label follow-on studies (HOPE/MTN-025 and DREAM) and other antiretroviral-based and multi-purpose prevention technology ring products advance through the development pipeline, there is a need for more accurate and reliable measures of adherence to microbicide ring products. We previously conducted a comprehensive landscape analysis to identify new technologies that could be applied to adherence measurement of vaginal rings containing antiretrovirals. To explore attitudes and perceptions towards the approaches that we identified, we conducted a survey of stakeholders with experience and expertise in microbicide and HIV prevention clinical trials. From May to July 2015 an electronic survey was distributed via email to 894 stakeholders; a total of 206 eligible individuals responded to at least one question and were included in the data analysis. Survey respondents were presented with various objective measures and asked about their perceived acceptability to trial participants, feasibility of implementation by study staff, usefulness for measuring adherence and ethical concerns. Methods that require no additional input from the participant and require no modifications to the existing ring product (i.e., measurement of residual drug or excipient, or a vaginal analyte that enters the ring) were viewed as being more acceptable to trial participants and more feasible to implement in the field. Respondents saw value in using objective measures to provide real-time feedback on adherence. However, approaches that involve unannounced home visits for sample collection or spot checks of ring use, which could provide significant value to adherence feedback efforts, were met with skepticism. Additional research on the acceptability of these methods to potential trial participants and trial staff is recommended.

## Introduction

Results of recent microbicide and pre-exposure prophylaxis (PrEP) clinical trials have shown participant adherence to be a significant challenge with new HIV prevention technologies [1–4]. Low levels of product use of oral and vaginal gel microbicides have largely impeded the ability to measure product efficacy [2, 4]. The use of vaginal rings for sustained delivery of antiretrovirals (ARV) has widely been viewed as a way to overcome adherence challenges shown with oral and vaginal gel products [5]. The recent Phase III trials of the vaginal ring containing the ARV dapivirine, ASPIRE (MTN-020) and The Ring Study (IPM 027), showed modest HIV prevention overall among women using the product [1, 6]. However, low levels of consistent use of the ring was cited as an issue in the trials, particularly among younger women, and subsequent analyses showed no detectable protection in participants with low adherence [7].

As the dapivirine ring product moves into two open label follow-on studies (HOPE/MTN-025 and DREAM) [8] and other ARV-based [9, 10] and multipurpose prevention technology rings [11, 12] in earlier stages of the development pipeline are progressing to clinical trials, the need for more reliable and objective measures of adherence of microbicide ring products is greater than ever. In the ASPIRE trial and The Ring Study, measurements of residual drug levels in returned rings and detection of drug in plasma were used to objectively measure ring use by participants [1]. While these measures provide useful information about product use, they also have key limitations. For example, both measures are only applicable to the study arm using the active ring product and do not allow for real-time adherence feedback to participants due to the need to preserve study blinding, and due to the time required for sample processing and analysis.

We recently conducted a comprehensive landscape analysis to identify and evaluate new biomarkers and biometric approaches to measure adherence to ARV-based vaginal ring products in clinical trials that may overcome limitations with existing approaches [13]. While we identified numerous methods that are currently being explored in PrEP and microbicide trials (e.g., hair drug analysis, dry blood spot drug detection), we also described other concepts that are still in the proof-of-concept stage or in early clinical studies. How well these newer methods may be received in the field by participants and study staff needs to be better understood before they are developed further and implemented in clinical trials. To explore attitudes and perceptions towards a number of the approaches that we identified, we conducted a survey of stakeholders with experience and expertise in microbicide and HIV prevention clinical trials. Our intent in surveying this population was to understand how stakeholders perceived and interpreted study participant perspectives and behavior, and the extent to which they would find various options for measuring and supporting adherence in clinical trials to be acceptable, feasible, useful and ethical when implemented in the field.

## Methods

### Study participants

From May to July 2015 an electronic survey was distributed via email to 894 stakeholders with experience and expertise in HIV prevention clinical trial design and implementation. The purpose of the survey was to assess stakeholders’ attitudes and perceptions toward various methods of adherence support and measurement in clinical trials involving ARV-based vaginal rings, including objective measures of adherence. Stakeholder contact information was gathered from publicly accessible websites and databases. Members of the major HIV prevention research networks (i.e., Microbicide Trials Network and HIV Prevention Trials Network), the Consortium for Ring Adherence and attendees of the 2014 HIV Research for Prevention (HIV R4P) meeting were specifically targeted.

The survey instrument was developed in Qualtrics. We established face validity with the tool by having the survey questions reviewed independently by internal and external colleagues with survey construction and content expertise. We conducted a limited pilot test with in-house colleagues who met eligibility requirements for the survey. The survey was sent out to the first group of stakeholders on May 18, 2015 via email. The email contained a short description of the purpose of the study followed by an electronic link to the survey itself. Two additional waves of recruitment emails were sent on June 1 and June 11, 2015. The survey remained open until July 6, 2015.

Inclusion criteria included being 18 years of age or older, having written English fluency, and having active involvement in the previous 24 months in at least one of the following roles or activities related to an ARV-based HIV prevention trial, or ancillary research directly related to such a trial, that enrolled women as participants: protocol team member; trial implementation staff (e.g., investigator, manager, coordinator); program officer from funding organization; adherence and product use counselor; trial participant recruiter; trial monitor; community liaison officer, outreach worker or educators; ethics review committee member or administrators; or ethics consultant. Of the 894 emails sent to stakeholders, 115 (13%) bounced back resulting in 780 emails that reached the intended individual. A total of 258 (29%) opened the link to the online survey and completed the eligibility questions and 225 (25%) met the eligibility requirements. Of those who were eligible, 219 (97%) consented to participate and 206 (92%) responded to at least one survey question and were included in the data analysis; 152 (68%) respondents completed the entire survey.

### Measures

Survey respondents were asked to give basic sociodemographic information including their gender, age, highest degree or level of education, geographic location and previous role(s) in HIV prevention clinical trials. Questions relating to biomarkers and biometric measures of adherence were based on findings from our previous landscape analysis. We asked stakeholders about several existing and hypothetical objective adherence measures including:

- use of a magnetometer to detect a magnet integrated into the vaginal ring for spot checks during unannounced home visits
- measurement of systemic absorption of a volatile taggant incorporated into the ring formulation using a breath test
- collection of hair, blood and vaginal fluid samples at random, unannounced home visits for analysis of drug content
- the use of an integrated electronic sensor or logger in the ring that could record continuous data on intravaginal temperature, pH, pressure or exposure to the external environment if the ring is removed
- measurement of residual drug levels in the ring once it is returned
- measurement of residual levels of a non-drug excipient that is either intrinsic to the ring's formulation or added to the ring
- measurement of accumulation of a known vaginal analyte in the ring once it is returned

After receiving a brief description of each of the adherence measures mentioned above, the stakeholders were asked to answer questions about their perceived acceptability to trial participants, feasibility of implementation by study staff, usefulness for measuring adherence, and ethics around use with trial participants. For some questions, stakeholders were asked to evaluate methods using a Likert scale-type format. In other instances, they were asked to rank the measures based on each of the criteria mentioned above or choose a certain number of methods that they felt most and least met the criteria. For some methods, follow-up questions were included about specific aspects of the technologies. Stakeholders were also asked about their views on providing participants feedback on their adherence and the likelihood of participants tricking or outsmarting the adherence measures during a trial.

### Data collection and analysis

Survey responses were compiled in Qualtrics. After data collection was completed, quantitative data were transferred to SPSS 17.0 for data cleaning and analysis. Categories for several variables, including age, geographic location and highest degree or level of education, were merged when cell counts were small and/or when categories were conceptually compatible. Three main categories of clinical trial roles were constructed based on the responses received: administrative/supervisory, field staff, and program officer/ethical reviewer. Administrative/supervisory roles include protocol team members and trial implementation staff (e.g., investigator, manager, coordinator); field staff include adherence and product use counselors, trial participant recruiters, and community liaison officers, outreach workers or educators; program officer/ethical reviewer roles include program officers at funding organizations, trial monitors, ethics review committee members/administrators and ethics consultants.

Chi-square tests were used to assess associations between categorical variables, where appropriate. In cases where expected frequencies were less than 5 for some crosstabulation cells, Fisher’s exact tests were used. All tests were assessed at the 5% significance level for two-sided comparisons.

Qualitative analysis was carried out on questions that allowed for open-ended responses. For each question with qualitative responses, one of three coders reviewed the responses and identified recurring and potentially significant themes. The three coders convened and discussed common themes and summarized findings from each question in a memo.

### Ethics

This study protocol was submitted to FHI 360’s Protection of Human Subjects Committee and was deemed exempt as it was determined that the study posed minimal risk to survey respondents. Potential survey respondents were informed via email about the purpose of the research, methods of stakeholder identification, that participation was voluntary, how confidentiality was going to be maintained, that risks were minimal and that there were no direct personal benefits for participating and who to contact for information on the research and their rights as study participants. Upon accessing the survey, the stakeholders were presented with a consent statement and eligibility screener. If interested and found to be eligible, they were given the option to accept or decline consent to be included in the study. Those who accepted consent were allowed to commence the survey.

## Results

### Respondent characteristics

Of the 894 emails sent to stakeholders, 115 (13%) bounced back resulting in 780 emails that reached the intended individual. A total of 258 (29%) stakeholders opened the link to the online survey and completed the eligibility questions and 225 (25%) met the eligibility requirements. Of the stakeholders who were eligible, 219 (97%) consented to participate and 206 (92%) responded to at least one survey question and were therefore included in the data analysis; 152 (68%) respondents completed the survey.

Most survey respondents (64.1%) were 40 years of age or older, 35% were between 25–39 years old and one respondent was between 18–24 years old (Table 1). Of the respondents who provided their gender, 74.3% were female and 25.7% male. Most respondents who provided their highest level of education reported having a master’s or doctoral degree (64.5%) and approximately a quarter had an undergraduate degree. Of the respondents who reported their geographic location, just over half were from Sub-Saharan Africa, 39% from North America, and the remaining 10% from South or Central America, the Caribbean, South or Southeast Asia, Europe or Australia. Nearly three-quarters of participants stated that they had an administrative or supervisory role in HIV prevention trials within the past 24 months, 35.4% reported having held a position in the field, and 14.6% were program officers for a funding organization or ethical reviewer.

**Table 1:** Respondent characteristics.

	n (%)
Sex (n = 152)	
Female	113 (74.3)
Male	39 (25.7)
Age (n = 206)	
18–24 years	1 (0.5)
25–39 years	73 (35.4)
40 years or older	132 (64.1)
Highest degree or level of education (n = 152)	
No college/some college/technical certificate	15 (9.9)
Undergraduate degree	39 (25.7)
Master’s level degree	52 (34.2)
Doctoral level degree	46 (30.3)
Geographic Location (n = 151)	
Sub-Saharan Africa	77 (51.0)
North America	59 (39.1)
South or Southeast Asia	6 (4.0)
South America/Central America/Caribbean	3 (2.0)
Europe or Australia	2 (1.3)
Other/not specified	4 (2.6)
HIV prevention clinical trial role (n = 206)^1^	
Administrative/supervisory staff	153 (74.3)
Field staff	73 (35.4)
Program officers/ethical reviewers	30 (14.6)

^1^Stakeholders were asked to indicate all roles held in the past 24 months so respondents may be included in multiple role categories

### Trial participant acceptability

**Fig 1:**
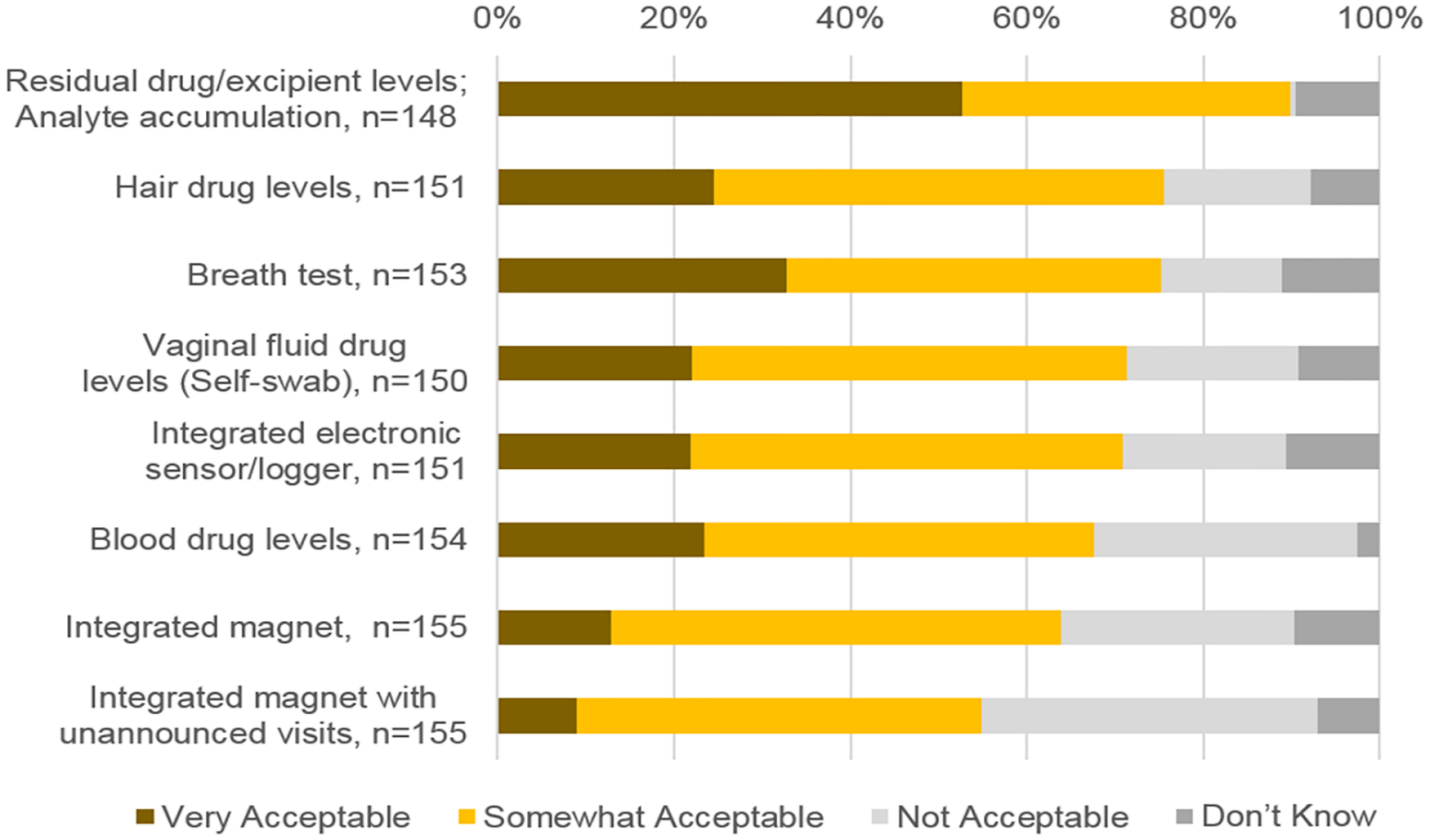
Respondents’ views on acceptability of measures for trial participants.

We first asked HIV prevention trial stakeholders about their views on the potential acceptability of various biometric measures of ring adherence to clinical trial participants. As shown in Fig 1, of the methods presented, analysis of returned rings for residual drug levels, measurement of depletion of an inactive ingredient out of the ring or measurement of a vaginal analyte that enters the ring during use were thought to be most acceptable to trial participants. Although these methods measure adherence differently, they were combined as one option for questions on acceptability as they would require the same level of input from the trial participant, which is simply to turn the ring over at their designated follow-up visit for analysis. Nearly 90% of respondents believed these approaches would be very acceptable or somewhat acceptable to women.

The use of a breath test to detect chemical taggants added to the ring formulation and the collection of hair samples for drug levels were also viewed as potentially being acceptable to women. Given past studies that showed the chemical taggants used for the breath test approach in vaginal gel products caused an unfavorable taste for some women [14, 15], survey respondents were asked an additional question about women’s acceptance of the method if they knew it would result in an unfavorable taste. Over three-quarters (78.6%) of respondents said that it was very likely or somewhat likely that women would refuse the method if this was the case (data not shown).

For the method of collecting and analyzing hair samples, we asked a follow-up question about whether respondents knew of any cultural barriers toward removal of 20–30 strands of hair for analysis, which is roughly the amount of hair needed to detect most ARVs in assays. Most of those who responded cited cultural beliefs as a factor that may prevent acceptance of hair removal. Witchcraft was frequently mentioned (59.4% of respondents) as a reason for why some women would oppose this method. In particular, many respondents mentioned Africans, Black Africans, and African cultures in general being opposed to this approach, and two respondents specifically reported people from the Zulu culture being opposed. Of those respondents who mentioned witchcraft as a barrier to uptake, several said that some people might be concerned with the disposal of hair once it is cut or removed from one’s head and that it would need to be disposed of properly (e.g., buried) to avoid the person becoming bewitched. Some respondents also cited women’s hair styles and hairdos, and their desire to protect them, as a factor that would make some women be opposed to giving hair samples. Similarly, it was reported that people with short hair might dislike this practice as bare spots may become noticeable over time.

The approach that was perceived to be least acceptable to women was the detection of a magnet integrated into the ring using a handheld magnetometer. Only 63.9% of respondents said that the method would either be very acceptable or somewhat acceptable to participants. Just over half (54.8%) of respondents believed this method would be very or somewhat acceptable if done in conjunction with unannounced home visits. In a follow-up question, 35.7% said that women would be very skeptical or worried and 53.2% would be somewhat skeptical or worried to have a magnet inside their bodies.

Further analysis showed that views on acceptability for hair sampling differed by respondent role and geographic location but not for other methods (data not shown). Those who had an administrative or supervisory role in HIV prevention trials in the past 24 months were more likely than those who did not to believe hair sampling would be very or somewhat acceptable to trial participants (86.6% vs. 63.0%; p = 0.009). Additionally, respondents from the Americas, Europe or Australia were marginally more likely to view hair sampling as being very or somewhat acceptable to trial participants than respondents from Sub-Saharan Africa (89.8% vs. 76.6%; p = 0.051); responses did not significantly differ between respondents in South or Southeast Asia and other areas of the world. Views on acceptability were not associated with respondent age, gender or education level for any adherence measure.

### Feasibility of implementation

**Fig 2:**
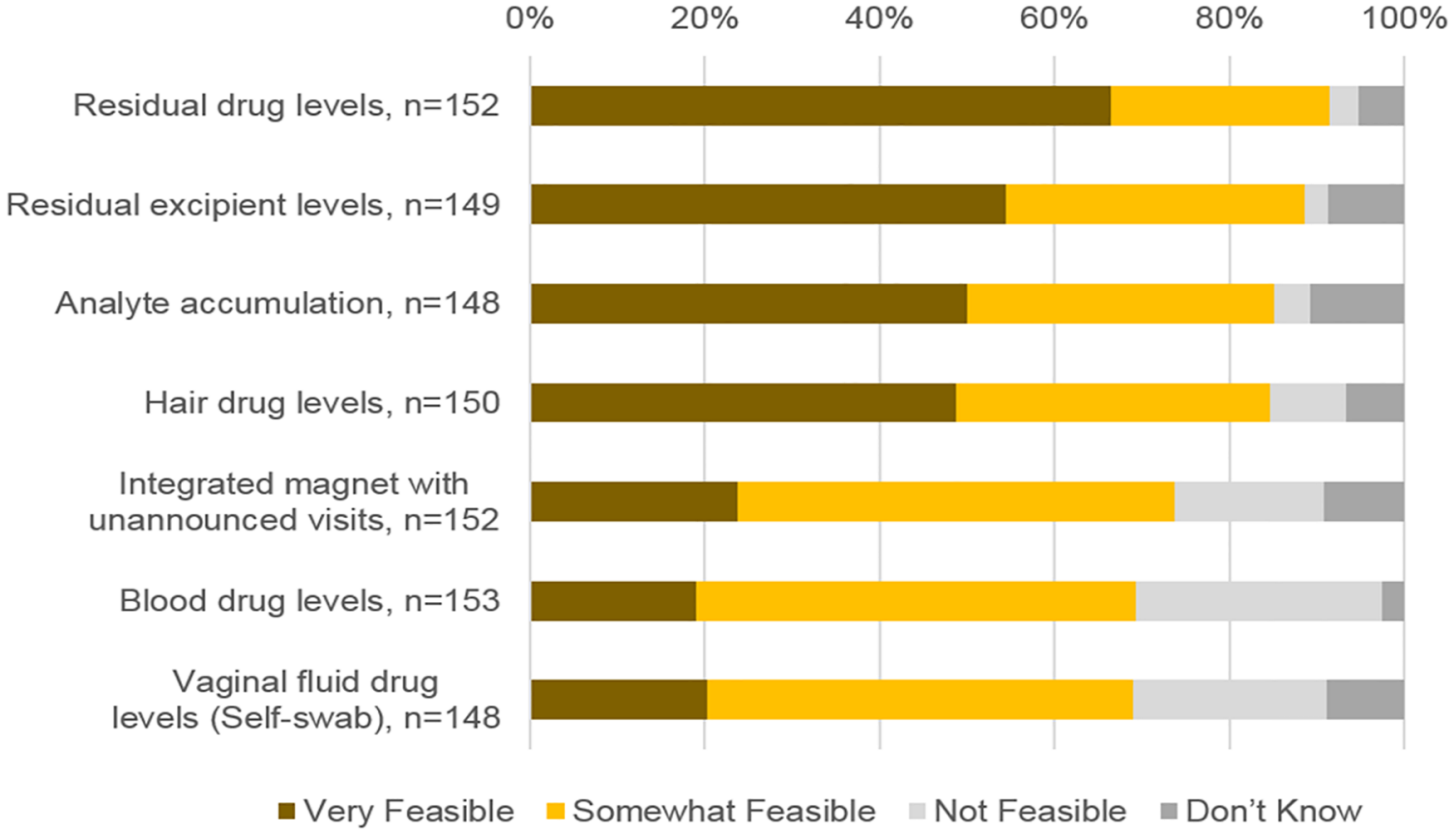
Respondents’ views on feasibility of implementing measures in clinical trials.

Stakeholders were also asked to give their opinion on how feasible implementation of various biometric measures would be in clinical trials. Like the findings on acceptability, analysis of returned rings for residual drug, a non-active excipient that diffuses out of the ring, or an analyte that enters the ring were viewed as being most feasible, with 91.4%, 88.6% and 85.1% of respondents indicating that these approaches would be very or somewhat feasible, respectively (Fig 2). Collection of hair samples was also seen as being quite feasible, with nearly 85% of respondents indicating that this approach would be very or somewhat feasible. Collection of blood samples, collection of vaginal fluid through self-swabbing and detection of rings using an integrated magnet at unannounced visits were viewed as being the least feasible. Of those who responded, 28.1%, 22.3% and 17.1% reported these methods would not be feasible, respectively.

Our analyses showed that views on the feasibility of hair collection as a measure of adherence was associated with respondent role and location. Respondents who held administrative or supervisory roles in HIV prevention trials were more likely to view hair collection as being very or somewhat feasible to implement in the field than those who did not (93.8% vs 77.8%; p = 0.02). Additionally, respondents who held a field-level position were less likely to view hair collection as very or somewhat feasible than those who had not (94.9% vs. 80.5%; p = 0.02). Respondents in America, Europe and Australia were more likely to view hair collection as being very or somewhat feasible than respondents in Sub-Saharan Africa (98.3% vs. 84.6%; p = 0.007). Respondents with more education (i.e., master’s or doctoral degree) were more likely to view blood collection as being very or somewhat feasible when compared to respondents with less education (i.e., undergraduate degree or less) (79.6% vs. 59.1%; p = 0.012). Views on feasibility were not associated with respondent age or gender for any adherence measure.

### Method usefulness

**Fig 3:**
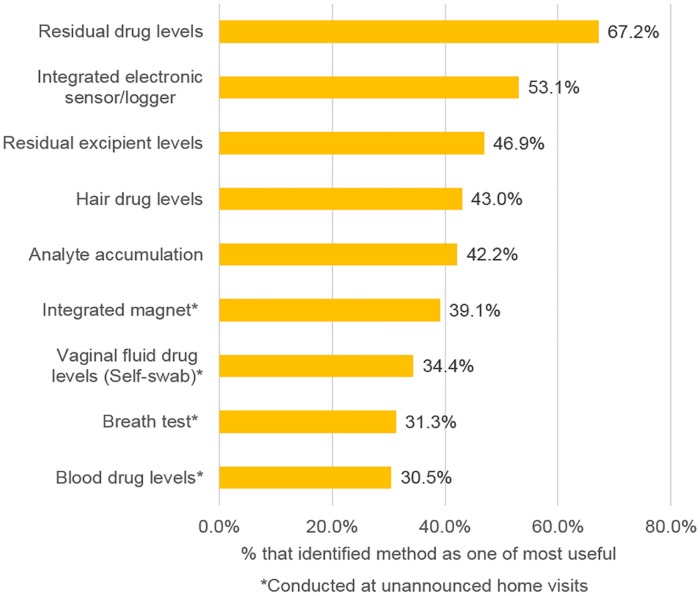
Respondents’ views on usefulness of measures for measuring adherence (n = 128).

Stakeholders were asked to identify which of the biomarkers and biometric approaches presented to them would be most useful for measuring vaginal ring adherence. As shown in Fig 3, measurement of residual drug in returned rings was viewed as being the most useful approach, with 67.2% of all respondents identifying it as one of the most useful approaches. The use of a ring with an integrated electronic sensor to make biometric measurements (e.g., temperature, pH, pressure monitoring) was also viewed as a highly useful approach. Those approaches that involved unannounced home visits were seen as being the least useful. Only 30.5%, 31.3%, 34.4% and 39.1% of respondents identified blood collection, breath tests, vaginal fluid collection and spot checks using a ring with an integrated magnet at unannounced home visits as one of the most useful measures of adherence, respectively.

### Ethical considerations

**Fig 4:**
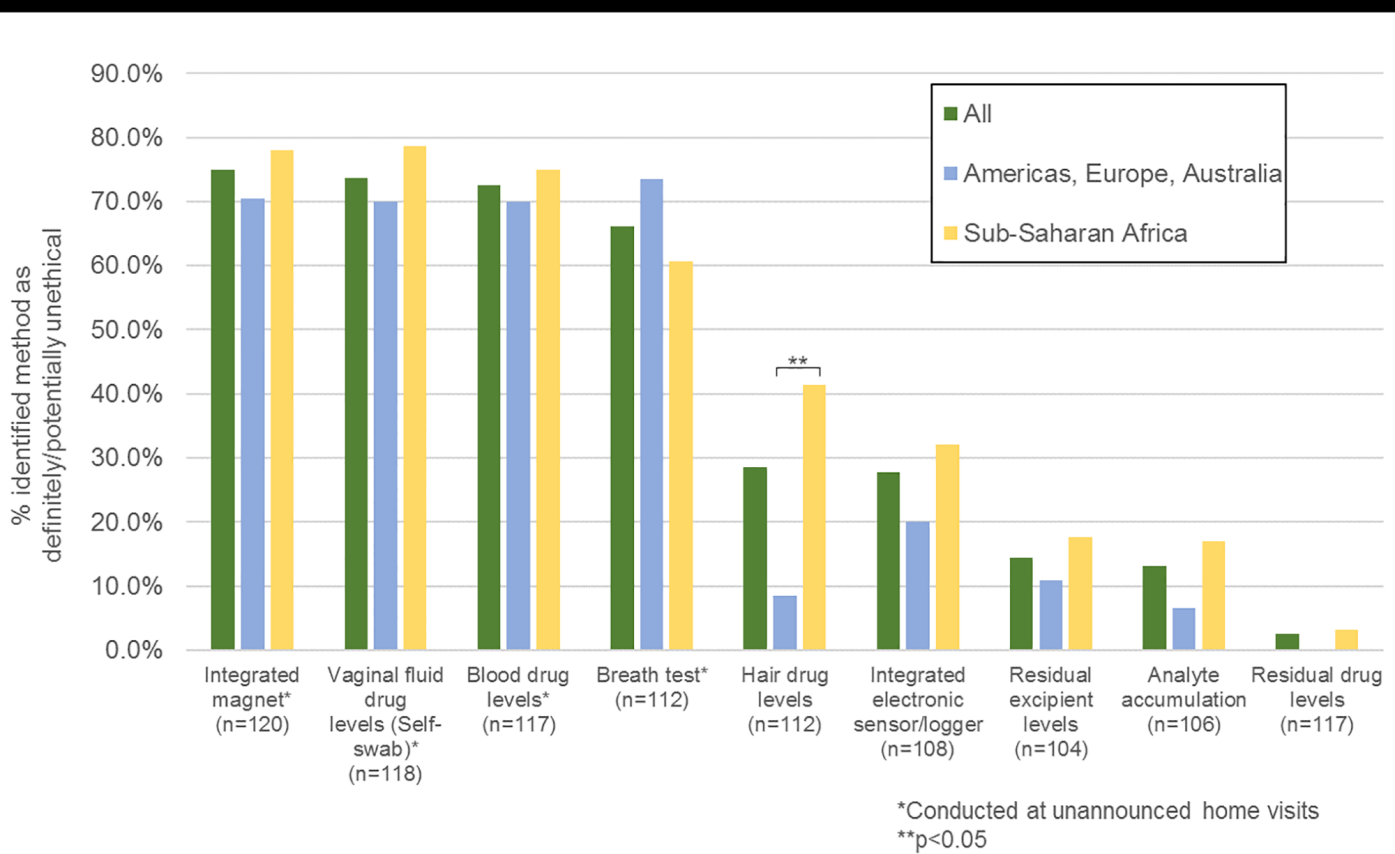
Ethical concerns for methods by region.

Survey respondents were asked to specify whether they believed each of the methods to be ethical, potentially ethical or definitely unethical to use in a clinical trial setting. The frequencies of respondents who showed some concern about the ethics of specific methods (i.e., either responded that the approach was potentially or definitely unethical) are shown in Fig 4. Respondents showed the least concern for measurement of residual drug, a non-active excipient that diffuses out of the ring, or a vaginal analyte that enters the ring. Many respondents indicated ethical concerns for the methods that involved unannounced home visits. The method viewed as least ethical was conducting spot checks of rings with an integrated magnet using unannounced home visits, with 67.9% indicating that the method was potentially unethical or definitely unethical.

Respondents’ views on the ethics of hair collection were shown to be associated with their geographic region and clinical trial role (data not shown). Respondents in Sub-Saharan Africa were more likely to think hair collection was potentially or definitely unethical when compared with respondents in the Americas, Europe or Australia (41.4% vs. 8.5%; p<0.001). Individuals with field-based positions were more likely to view hair collection as potentially or definitely unethical than those who did not have field-based roles (43.2% vs. 22.1%; p = 0.02) whereas individuals with an administrative/supervisory role were more likely to see hair sample collection as being an ethical approach (78.5% vs. 38.1%; p<0.001) when compared to those who had not held an administrative/supervisory role. Individuals with an administrative/supervisory role were also more likely to view measurements of analyte accumulation into the ring as ethical (91.0% vs. 70.0%; p = 0.021). Finally, younger respondents (i.e., 18–39 years of age) were more likely say that detection of a magnetic ring during unannounced home visits would be potentially or definitely unethical than older respondents (i.e., 40+ years of age) (86.4% vs. 69.2%; p = 0.035). Views on ethics were not associated with respondent gender or education for any adherence measure.

### Trial participant adherence feedback

Some of the biometric measures identified in our previous landscape analysis allow for real-time feedback on adherence, including the use of rings with integrated magnets or RFID tags to conduct spot checks or examining the color of worn rings. When asked whether participants should be given feedback about their adherence during a trial based on biomarker or biometric results, responses were positive with 84.7% of respondents agreeing with this statement. When stakeholders were asked about what they thought to be an appropriate frequency to provide adherence feedback to trial participants, 50.3% of those surveyed said at every follow-up visit (the typical schedule of follow-up visits has been monthly in the context of biomedical HIV prevention trials, including those of vaginal rings) and 38.5% said 2–4 times per year. Only 6.3% said that counselors should not give participants feedback on adherence based on objective measures. Additionally, of all survey respondents, 95.8% indicated that trial staff would find providing feedback to women very acceptable or somewhat acceptable and 95.1% indicated that trial participants would find receiving feedback very acceptable or somewhat acceptable (data not shown).

### Method manipulation

**Fig 5:**
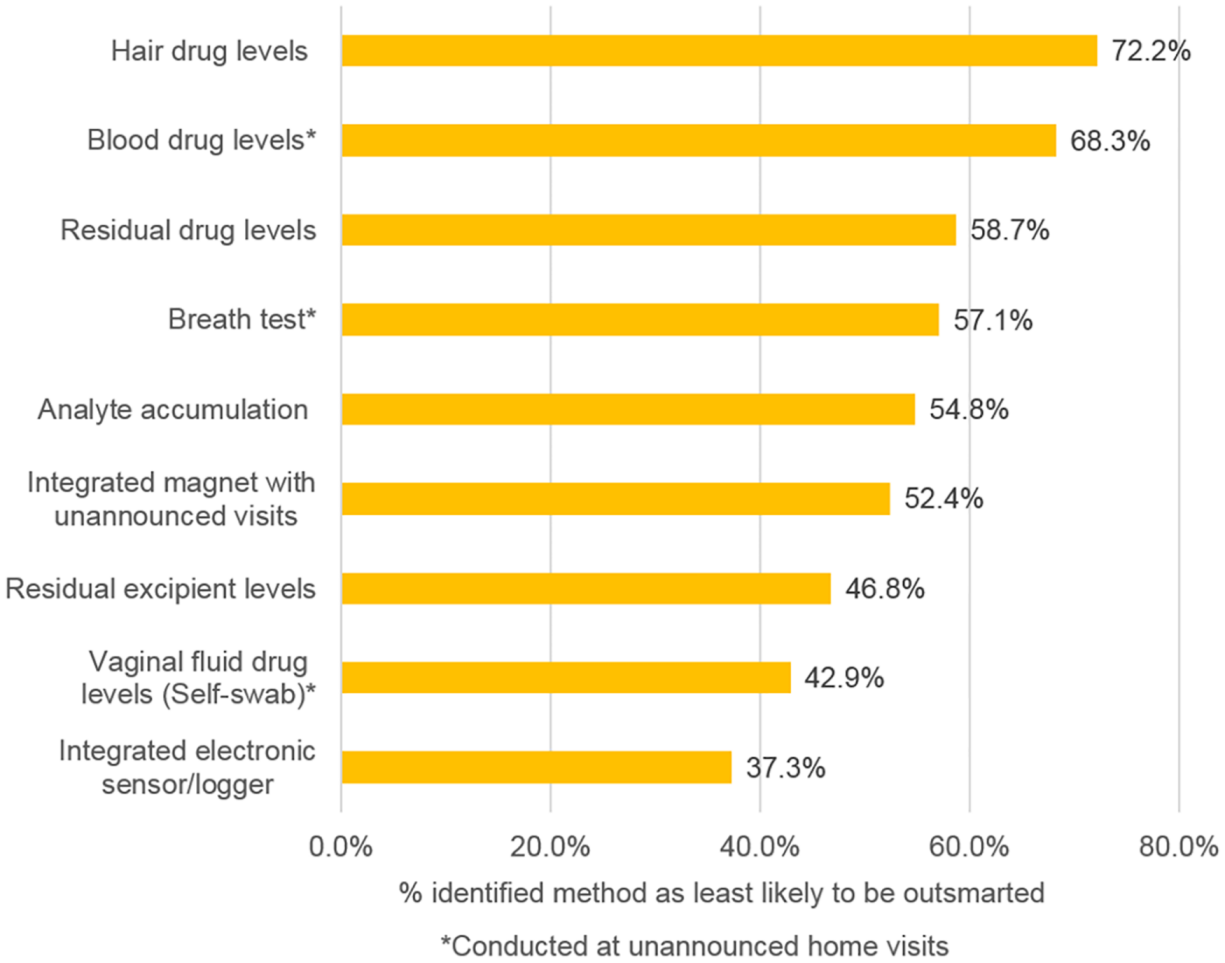
Measures identified as being less prone to being tricked or outsmarted (n = 126).

Finally, respondents were asked whether the proposed measures would be susceptible to being tricked or outsmarted by trial participants. Generally, those who responded were doubtful that trial participants would try to trick or outsmart adherence measures. Only 8% said many or most participants would try to outsmart these measures while 46% said that some but not most participants would attempt it (data not shown). However, stakeholders identified certain biometric measures as being more susceptible to manipulation than others should participants try to do so. Rings with integrated electronic sensors and collection of vaginal fluid via self-swabs were thought to be most susceptible to being outsmarted (Fig 5). Hair sample drug analysis, measurement of blood drug levels and residual drug measurements were seen as the three methods that were least likely to be outsmarted. Further analysis showed that views on participant’s likelihood to manipulate adherence measures did not vary by respondent age, gender, education, location or role (data not shown).

## Discussion

A better understanding of women’s adherence to ARV-based vaginal rings is critical for assessing product effectiveness in future clinical trials. We previously conducted a landscape analysis to identify new and existing technologies that can be applied toward measurement of vaginal ring adherence [13]. With this survey, we aimed to obtain valuable formative information about HIV prevention stakeholders’ views on the implementation of many of the measures identified in this landscape analysis.

Measures that were viewed by stakeholders to be most acceptable to participants and feasible for clinical trial staff to implement were measurements of residual drug in the ring, a vaginal analyte that enters the ring during use and depletion of a non-drug excipient out of the ring. This finding is not surprising considering that these approaches are non-intrusive and require no additional input from participants than existing procedures. Sample collection simply involves retrieving the ring from the participant at designated follow-up visits. These methods may also be viewed more favorably because no alterations to the existing ring product are required. Women may take issue with use of rings that include electronics, magnets or other unknown entity. Furthermore, from a clinical trial management perspective, modifying the ring may lead to additional costs and regulatory and ethical issues.

Stakeholders believed the measurement of residual drug to be the most useful followed by use of an electronic sensor in the ring and the measurement of depletion of a non-drug excipient from the ring. Measurement of residual drug has been explored in the clinical trials of the dapivirine ring, with analyses showing a decrease in drug concentration after longer periods of use [16]. A major disadvantage of this method, however, is that it can only be used with the arm of the trial using the active ring product. Therefore, as long as a placebo ring is required for use with a comparison group, this method will exclude a portion of the study sample, and raises the potential for bias in the event that adherence behaviors correlate with risk taking behaviors. Because of this, we recommend exploring methods to measure the diffusion of a vaginal analyte into the ring, and methods to measure depletion of a non-drug excipient out of the ring, as these approaches can be applied to both active and placebo ring products [13, 17, 18].

Collection of hair samples was also viewed by stakeholders as being acceptable to participants, feasible to implement, useful to measure adherence and being at the lowest risk for participants manipulating the method. We have previously identified this method as being of high priority for further development due to the ease of sample collection, transport and storage and strong correlations shown between hair drug levels and oral doses of tenofovir [19] and other adherence measures [20, 21]. Additionally, novel assays aimed at lowering the cost of hair sample analysis [22] and providing higher resolution quantification of drug levels [23] are in development.

While these findings from the stakeholders are promising, it is important to acknowledge that acceptability of hair collection will likely vary between different populations and settings. Stakeholders from Sub-Saharan Africa in our survey seemed to perceive this approach as being less acceptable to trial participants than stakeholders in the Americas, Europe and Australia. Other studies in Kenya and Uganda have reported ≥95% participant acceptability of the method [20, 24], while qualitative data from South Africa shows participants may be reluctant to donate hair due to fear of the hair being used in witchcraft or other means to inflict harm [25]. A study involving hair collection from children in rural Uganda proved to be challenging due to participants declining sample collection or already having their heads clean-shaven [26]. Therefore, it is important that further research on the acceptability of hair collection be done, especially in populations where trials will be conducted, and community sensitization and education take place to dispel myths (e.g., its potential use for witchcraft) and maximize uptake during a trial.

Our results suggest that the stakeholders see value in providing participants with real-time feedback on adherence using biometric measures and that participants and trial staff may be welcoming to this approach. Unfortunately, the methods that are the most useful for facilitating the provision of real-time feedback, i.e., the approaches that involve unannounced home visits for sample collection or spot checks of ring use, were met with overwhelming skepticism. Not only were approaches involving unannounced home visits seen as being problematic from acceptability, feasibility and ethical standpoints, but very few stakeholders saw them as being useful adherence measures. Follow-up questions were not asked about the specific issues stakeholders had with these approaches, but we speculate that their concerns with unannounced visits lie with the potential invasion of privacy, inadvertent ‘outing’ of women as HIV prevention trial participants and/or, in the case of the use of an integrated magnet, concerns about the safety of the device. Evidence from recent HIV prevention trials has shown women may avoid disclosing study participation to their partner or members of their community out of fear of negative reactions (e.g., partner disapproval or discrimination by peers), questioning or misattribution of participation to having an HIV-positive status [27–31]. Provision of educational materials such as information leaflets or opportunities for direct contact with investigators have been suggested as means to improve partner and community understanding about the study and product [28], which may help to assuage participant concerns about ‘outing’ from adherence measures. Motivational support for product adherence combined with targeted support for identifying and implementing behaviors that facilitate adherence also show evidence of effectiveness [32]. Counseling about the safety and risks of any new or foreign technology used in the ring to monitor use will also be critical to maximize product uptake and adherence.

Despite these findings, we still believe it would be a worthwhile effort to collect additional data on the acceptability of unannounced visits as this approach would add tremendous value to adherence support efforts. Previous research has shown that unannounced pill counts, while resource intensive, can be an effective approach to monitoring adherence in clinical studies [33–35]. Until more research is done, it would be premature to conclude that this would be infeasible when used with other adherence measures.

We acknowledge some potential limitations with this study. First, the use of an online survey is susceptible to selection bias as it is limited to those with internet access during the timeframe the survey was open and those whose contact information was available in the databases searched. As only 206 (23%) of the 894 individuals who were invited to participate in the survey were included in data analyses and approximately nine in ten respondents were from North America or Sub-Saharan Africa, results may not be representative of the entire HIV prevention trial field. Additionally, stakeholders’ views on acceptability may not reflect the attitudes and perceptions of actual trial participants. Therefore, additional acceptability research involving past or potential trial participants is needed. Also, while we did provide descriptions of the biometric methods presented in the survey, we were not able to clarify details or answer questions respondents may have had. Therefore, questions may have been answered with incomplete or inaccurate understanding about the specific technologies.

Our results show that vaginal ring adherence measures that require no additional input from the participant and require no modifications to the existing ring product (e.g., measurement of residual drug or excipient, or a vaginal analyte that enters the ring) are viewed by HIV prevention trial stakeholders to be most likely to be accepted by trial participants and will be most feasible to implement in the field. Collection of hair samples was also seen to bring value to trials involving ARV-based rings but views differ by respondents’ geographic location and role in clinical trials. Methods that involve unannounced home visits are seen as being most problematic and raise ethical concerns. However, given their value in providing real-time adherence feedback to participants, additional research on methods that involve unannounced visits may still be worthwhile. We recommend further investigation of these approaches to fill the gaps in existing adherence measures and allow for more accurate appraisals of vaginal ring use.

## Supporting information

S1 File Relevant survey questions. (PDF)
