# Supplementary material for: Attitudes and perceptions towards novel objective measures of ARV-based vaginal ring use: Results from a global stakeholder survey
Source: PLoS One. 2017 Jul 14;12(7):e0180963. doi: 10.1371/journal.pone.0180963 (PMC5510854; doi:10.1371/journal.pone.0180963)
Supplement: S1 File — (PDF) [file pone.0180963.s001.pdf]

Q2.1 First we'd like to ask a few questions to determine if you are eligible to participate in this study.   How old are you?

- ❍ Less than 18 years (1)
- ❍ 18-24 years (2)
- ❍ 25-39 years (3)
- ❍ 40 years or older (4)

Q2.2 Do you have fluency in written English?

- ❍ Yes (1)
- ❍ No (2)

Q2.3 In the previous 24 months, have you been actively involved in any of the following roles or activities related to an ARV-based HIV prevention trial, or ancillary research directly related to such a trial, that enrolled women as participants? Please select all that apply.

- ❑ Protocol team member (1)
- ❑ Trial implementation (e.g., investigator, manager, coordinator) (2)
- ❑ Program officer at a funding organization (3)
- ❑ Adherence and product use counselor (4)
- ❑ Trial participant recruiter (5)
- ❑ Trial monitor (6)
- ❑ Community liaison officer, outreach worker, or educator (7)
- ❑ Ethics review committee member or administrator (8)
- ❑ Ethics consultant (9)
- ❑ None of the above (10)

[If Q2.1=1, Q2.2=2 OR Q2.3=10] Q3.1 We're sorry, but you do not meet the eligibility requirements for this survey. We thank you for your interest and appreciate your time.

[If Q2.1=2,3,4 AND Q2.2=1 AND Q2.3=1,2,3,4,5,6,7,8,9] Q4.1 Thank you for answering these questions. You meet the eligibility criteria for this study. Do you consent to participate in this survey?

- ❍ Yes, I consent to participate in this survey. (1)
- ❍ No, I do not consent to participate in this survey. (2)

Answer If Q4.1=1

Q4.2 Some of the questions in the survey will be about vaginal rings. Here is a picture of a vaginal ring made of silicone that includes an antiretroviral called dapivirine. It is currently being tested in phase III clinical trials in Africa by the International Partnership for Microbicides (IPM) and the Microbicide Trials Network (MTN). Other ARV-based rings, including some with a contraceptive drug, are in development.

Answer If Q4.1=1

Q4.3

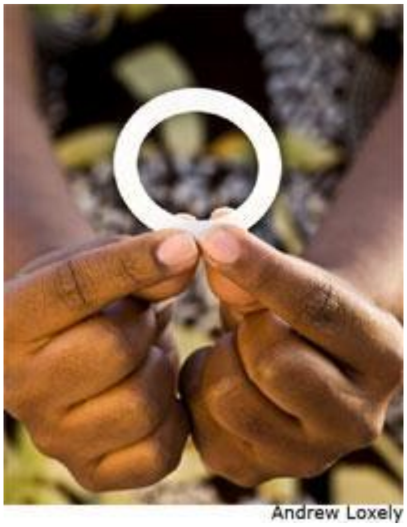

Answer If Q4.1=1

Q4.4 Please note that while taking this survey, you can stop at any time and return to continue the survey later -- your responses are automatically saved as you go along. By using the arrow buttons at the bottom of the page, you may scroll back and forth within most sections, but you will not be able to page backwards between different sections.

Q16.1 Biometric Technologies & Approaches to Detect Adherence In this set of questions we will describe several biometric technologies or methods that could be used to objectively measure vaginal ring adherence in clinical trials. Based on your experiences and local knowledge, we would like to know if you think these biometric technologies and methods would be acceptable to research teams and women who might participate in HIV prevention trials in the settings where you work.

Q17.1 Magnets embedded in vaginal rings One technology being considered to detect and measure vaginal ring usage is the inclusion of a very small magnet (magnetic tag) embedded in a vaginal ring. Magnetic tags emit a magnetic field that could be detected up to several feet, depending on the strength of the magnet. A magnetic tag could be as small as a pinhead and potentially invisible when placed in the ring. Portable handheld meters can detect and measure magnets, are readily available, and could be used by trial staff with minimal training to detect and measure the presence or absence of a ring in a woman’s vagina. Use of a handheld meter would not require a physical exam, or physically touching the woman in order to

determine if the ring was inserted in the vagina. For example, the meter would be able to tell the difference between a ring placed in a skirt pocket and one inserted in the vagina.

Q17.2 In your opinion, how acceptable to women participating in clinical trials would it be to use a ring embedded with a magnetic tag for detecting and measuring vaginal ring adherence in clinical trials?
- Very acceptable (1)
- Somewhat acceptable (2)
- Not acceptable (3)
- Don't know (4)

Q17.3 Do you think women would be skeptical or worried about having a very small magnet in their bodies?
- Yes, they would be very skeptical or worried (1)
- Yes, they would be somewhat skeptical or worried (2)
- No, they would not be skeptical or worried (3)
- Don't know (4)

Q17.4 In general, do you think women would prefer a physical (vaginal) exam or use of a hand-held meter to detect the presence of a ring embedded with a magnetic tag within the vagina?
- Women would prefer use of a meter and ring with magnetic tag to check for ring insertion (1)
- Women would prefer a physical (vaginal) exam to check for ring insertion (2)
- Women would have no preference for either using a meter and ring with magnetic tag or a physical (vaginal) exam (3)
- Don't know (4)

Q17.5 One potential advantage of using magnetic tags is that trial staff could potentially check for ring adherence at home visits without having to do a physical (vaginal) exam. In your opinion, how acceptable do you think it would be to trial staff themselves to use a meter at home visits to measure real-time adherence of vaginal rings embedded with magnetic tags?
- Very acceptable (1)
- Somewhat acceptable (2)
- Not acceptable (3)
- Don't know (4)

Q17.6 In your opinion, how feasible or practical do you think it would be for trial staff to carry out real-time adherence checks of trial participants with a meter at home visits to measure real-time adherence of vaginal rings embedded with magnetic tags?
- Very feasible (1)
- Somewhat feasible (2)
- Not feasible (3)
- Don't know (4)

Q17.7 Spot checks of vaginal rings with magnetic tags could be carried out at random, unannounced home visits. At the beginning of the trial, in the informed consent process, women would be asked to consent, or agree, to having random, unannounced home visits that might include checking for ring adherence with a hand-held meter, and they would be informed about whether and how often these unannounced home visits might occur during the trial.   In your opinion, how acceptable to participants do you think it would be to use random, unannounced, home visits for the purpose of performing spot checks on ring insertion via use of a hand-held meter?

- Very acceptable (1)
- Somewhat acceptable (2)
- Not acceptable (3)
- Don't know (4)

Q18.1 Breath test - volatile tagents Another possible biometric technology centers on the use of a breath test to detect the presence of a non-toxic chemical added to the vaginal ring. The chemical would be absorbed by the body in a way that would allow it to be detected using the breath test. Trial participants would be asked to blow into a plastic syringe or bag using a mouthpiece. The air would then be tested to see if the chemical from the ring is present. If the chemical is not present, it means that the ring was removed from the vagina. This method has been piloted with microbicidal gels, but the technology has not yet been developed for vaginal rings. In general, how acceptable to women participating in clinical trials would it be to take a breath test to detect the presence of a vaginal ring?

- Very acceptable (1)
- Somewhat acceptable (2)
- Not acceptable (3)
- Don't know (4)

Q18.2 The chemical used in the microbicidal gel pilot resulted in an unacceptable taste for some, but not all, participants. If women knew they may experience an unfavorable taste from a chemical used in a vaginal ring, how likely do you think it is that women would refuse to use the ring?

- Very likely (1)
- Somewhat likely (2)
- Not likely (3)
- Don't know (4)

Q19.1 Collection of hair, blood, and vaginal fluid samples Another type of approach to assessing adherence would be to collect blood, vaginal fluid, or hair samples from study participants. Participants would be told how, when, and where such samples would be collected during the informed consent process.

Q19.2 Blood samples Drug levels in the blood could be used to objectively assess adherence to the vaginal ring, as has been done in several other microbicide and PrEP clinical trials. This would involve either a traditional blood draw with a needle stick or a finger prick for dried blood

spot collection. How acceptable do you think collecting blood samples at random, unannounced home visits would be to trial participants, in order to detect and measure product adherence?

- ❍ Very acceptable (1)
- ❍ Somewhat acceptable (2)
- ❍ Not acceptable (3)
- ❍ Don't know (4)

Q19.3 How feasible or practical would it be for trial staff to collect blood samples at random, unannounced home visits with participants?

- ❍ Very feasible (1)
- ❍ Somewhat feasible (2)
- ❍ Not feasible (3)
- ❍ Don't know (4)

Q19.4 Vaginal Fluid samples Collecting vaginal fluid samples to measure drug levels may be seen as more physically intrusive than collecting blood because of the nature of the collection procedure. One way to reduce the intrusiveness is for study participants themselves to self-swab while under a privacy gown. This approach could be used during random, unnanounced home-checks. This method, self-insertion of vaginal swabs with use of a privacy gown, has been successfully used in some studies to measure product adherence.

	Very (1)	Somewhat (2)	Not at all (3)	Don't know (4)
How acceptable to study participants would it be to have women self-swab for vaginal fluid in privacy at random, unannounced home visits? (1)	❍	❍	❍	❍
How feasible or practical do you think it would be to have women self-swab for vaginal fluid at random, unannounced home visits? (2)	❍	❍	❍	❍

Q19.5 In general, do you think women would prefer a physical (vaginal) exam to collect vaginal fluid or to self-swab for vaginal fluid during a home visit?
- ❍ Women would prefer to self-swab for vaginal fluid (1)
- ❍ Women would prefer a physical (vaginal) exam to collect vaginal fluid (2)
- ❍ Women would have no preference for either a self-swab for vaginal fluid or a physical (vaginal) exam (3)
- ❍ Don't know (4)

Q19.6 Hair samples Another approach would be the collection of hair samples to detect drug levels in the hair. This would involve taking about 20 to 30 strands of hair from a participant and testing it in the lab. The collection of hair samples could take place during regular clinic follow-up visits.

	Very (1)	Somewhat (2)	Not at all (3)	Don't know (4)
How acceptable to study participants would it be to take a hair sample of about 20 to 30 strands to check drug levels? (1)	❍	❍	❍	❍
How feasible do you think it would be for trial staff to collect hair samples during clinic visits to assess drug levels? (2)	❍	❍	❍	❍

Q19.7 Do you know of any cultural practices or specific populations that would tend to be opposed to the removal of 20 or 30 strands of a woman's hair? If so, please describe.
- ❍ Yes (1) ____________________
- ❍ No (2)
- ❍ Don't know (3)

Q20.1 Electronic Sensor measures Another approach to measuring vaginal ring adherence over time would be to place a very small electronic sensor inside a ring to measure certain kinds of exposures. Examples include: A sensor that measures how long a ring is exposed to air. Since the ring would have to be removed in order for it to be exposed to air, the device would indicate whether and how long a ring was removed. A temperature sensor that measures how long a ring is exposed to internal body temperatures; this would indicate whether and how long a ring was inserted in the vagina. A pressure sensor that can tell whether and how long a ring is

inserted in the vagina. A pH sensor that is calibrated to the pH level of the vagina and can tell how long a ring is inserted.

Q20.2 In your opinion, how acceptable to women participating in clinical trials would it be to use an electronic sensor embedded in a vaginal ring to measure adherence through things like exposure to temperature, pH, air, or vaginal pressure?
- ❍ Very acceptable (1)
- ❍ Somewhat acceptable (2)
- ❍ Not acceptable (3)
- ❍ Don't know (4)

Q20.3 Do you think women would be skeptical or worried about having a vaginal ring with this kind of sensor in their bodies?
- ❍ Yes, they would be very skeptical or worried (1)
- ❍ Yes, they would be somewhat skeptical or worried (2)
- ❍ No, they would not be skeptical or worried (3)
- ❍ Don't know (4)

Q20.4 It is possible that some trial participants would try to outsmart this approach by removing their rings and storing them in ways that might "fool" the sensor. How likely do you think it is that trial participants would be able to outsmart an electronic sensor that measures things like exposure to temperature, pH, air, or vaginal pressure?
- ❍ Very likely (1)
- ❍ Somewhat likely (2)
- ❍ Not likely (3)
- ❍ Don't know (4)

Q21.1 Measurement of residual drug in used rings Another approach is to require that trial participants return their used rings to study staff at the end of the ring's use period so it can be evaluated to see how much drug is left in the ring. This method can determine whether drug has been released while being worn in the vagina. It cannot measure the number of days a ring was used, but if the amount of drug in the ring is similar to that in an unused ring, it means that the ring probably was not worn at all or not worn long enough to provide protection. If placebo rings are used during a trial, they could not be used in this way to measure adherence because there would be no drug to assess. Therefore, to maintain blinding, this approach could not be used to determine which participants are adherent until after a trial had ended, but it could be used to get an idea of overall levels of adherence among participants at a given site during the trial.

	Very (1)	Somewhat (2)	Not at all (3)	Don't know (4)
How feasible or practical do you think measuring residual drug levels in vaginal rings would be for detecting and objectively measuring vaginal ring adherence in clinical trials? (2)	❍	❍	❍	❍
How useful do you think measurement of residual drug in rings would be for assessing adherence in clinical trials? (3)	❍	❍	❍	❍

Q22.1 Diffusion of analytes into or out of the ring It may also be possible to estimate how long a vaginal ring has been used by adding an inactive substance to the ring that would diffuse out of the ring when it is inserted in the vagina, just like the active drug in the ring can diffuse out. The inactive substance would be a material that is known to be safe when used in the body in this way. The advantage to this approach is that such a substance could be added to the placebo ring. Just as with the active drug, the amount of this substance left in the ring could be measured to determine if the ring had been worn.

	Very (1)	Somewhat (2)	Not at all (3)	Don't know (4)
How feasible or practical do you think measuring diffusion of an inactive substance out of the ring would be for detecting and objectively measuring vaginal ring adherence in clinical trials? (1)	❍	❍	❍	❍
How useful do you think measuring diffusion of an inactive substance out of the ringwould be for assessing adherence in clinical trials? (2)	❍	❍	❍	❍

Q22.2 Another approach would be to look for a substance already found in the vagina that can diffuse into the ring. For example, glycogen and cholesterol are substances that are present in the vagina and could potentially diffuse into the ring over time. The amount of the substance in the ring could be measured in the lab after the ring is returned. This kind of measure could be used with both active and placebo rings, and therefore could be used to determine which participants are adherent during the trial.

	Very (1)	Somewhat (2)	Not at all (3)	Don't know (4)
How feasible or practical do you think measuring diffusion of a vaginal substance into the ring would be for detecting and objectively measuring vaginal ring adherence in clinical trials? (2)	❍	❍	❍	❍
How useful do you think measuring diffusion of a vaginal substance into the ring would be for assessing adherence in clinical trials? (4)	❍	❍	❍	❍

Q22.3 In your opinion, how acceptable to women participating in clinical trials would it be to use measurement of residual drug levels or substances that diffuse into or out of vaginal rings to assess adherence during a clinical trial?

❍ Very acceptable (1)
❍ Somewhat acceptable (2)
❍ Not acceptable (3)
❍ Don't know (4)

Q23.1 Ranking of Biometric technologies and approaches We would like to ask you to rank the various biometric technologies and approaches for acceptability, feasibility, and usefulness below. If needed to aid in memory, a reference document with brief summaries of the various technologies and approaches can be accessed here: Biometric technologies reference.

Q23.2 We would like to know which biometric technologies and approaches you think trial participants would find the most and the least acceptable. Please choose the three most acceptable and drag them to the top box, and choose the three least acceptable, and drag them to the bottom box.

MOST ACCEPTABLE approaches to ring trial participants (choose 3)	LEAST ACCEPTABLE approaches to ring trial participants (choose 3)
______ Magnetic tag in ring and hand-held meter during unannounced home visits (1)	______ Magnetic tag in ring and hand-held meter during unannounced home visits (1)
______ Breath test for chemical in ring during unannounced home visits (4)	______ Breath test for chemical in ring during unannounced home visits (4)
______ Measurement of residual drug in returned ring (6)	______ Measurement of residual drug in returned ring (6)
______ Sensor to measure exposure to temperature, air, pH, or vaginal pressure in returned ring (7)	______ Sensor to measure exposure to temperature, air, pH, or vaginal pressure in returned ring (7)
______ Diffusion of vaginal substance into returned ring (9)	______ Diffusion of vaginal substance into returned ring (9)
______ Diffusion of safe, inactive substance out of returned ring (10)	______ Diffusion of safe, inactive substance out of returned ring (10)
______ Collecting hair samples during clinic visits (12)	______ Collecting hair samples during clinic visits (12)
______ Collecting blood samples at unannounced home visits (13)	______ Collecting blood samples at unannounced home visits (13)
______ Collecting vaginal fluid sample by self-swab during unannounced home visits (14)	______ Collecting vaginal fluid sample by self-swab during unannounced home visits (14)

Q23.3 We would like to know which technologies and approaches you think would be most feasible and practical. Please choose three that you feel are the most feasible and drag them to the top box, and choose three that you would find least feasible, and drag them to the bottom box.

MOST PRACTICAL approaches to measure adherence (choose 3)	LEAST PRACTICAL approaches to measure adherence (choose 3)
______ Magnetic tag in ring and hand-held meter during unannounced home visits (1)	______ Magnetic tag in ring and hand-held meter during unannounced home visits (1)
______ Breath test for chemical in ring during unannounced home visits (4)	______ Breath test for chemical in ring during unannounced home visits (4)
______ Measurement of residual drug in returned ring (6)	______ Measurement of residual drug in returned ring (6)
______ Sensor to measure exposure to temperature, air, pH, or vaginal pressure in returned ring (7)	______ Sensor to measure exposure to temperature, air, pH, or vaginal pressure in returned ring (7)

______ Diffusion of vaginal substance into returned ring (9)	______ Diffusion of vaginal substance into returned ring (9)
______ Diffusion of safe, inactive substance out of returned ring (10)	______ Diffusion of safe, inactive substance out of returned ring (10)
______ Collecting hair samples during clinic visits (12)	______ Collecting hair samples during clinic visits (12)
______ Collecting blood samples during unannounced home visits (13)	______ Collecting blood samples during unannounced home visits (13)
______ Collecting vaginal fluids through self-swabbing during unannounced home visits (14)	______ Collecting vaginal fluids through self-swabbing during unannounced home visits (14)

Q23.4 We are also interested in finding out your opinions on the usefulness of these various approaches and technologies. Please choose the items that you feel are the most useful and drag them to the top box, and choose the ones that you would find least useful, and drag them to the bottom box. You can select as many items from the list as you like for each category.

MOST USEFUL approaches to measure adherence	LEAST USEFUL approaches to measure adherence
______ Magnetic tag in ring and hand-held meter during unannounced home visits (1)	______ Magnetic tag in ring and hand-held meter during unannounced home visits (1)
______ Breath test for chemical in ring during unannounced home visits (4)	______ Breath test for chemical in ring during unannounced home visits (4)
______ Measurement of residual drug in returned ring (6)	______ Measurement of residual drug in returned ring (6)
______ Sensor to measure exposure to temperature, air, pH, or vaginal pressure in returned ring (7)	______ Sensor to measure exposure to temperature, air, pH, or vaginal pressure in returned ring (7)
______ Diffusion of vaginal substance into the returned ring (9)	______ Diffusion of vaginal substance into the returned ring (9)
______ Diffusion of safe, inactive substance out of returned ring (10)	______ Diffusion of safe, inactive substance out of returned ring (10)
______ Collecting hair samples during clinic visits (12)	______ Collecting hair samples during clinic visits (12)
______ Collecting blood samples during unannounced home visits (13)	______ Collecting blood samples during unannounced home visits (13)
______ Collecting vaginal fluid samples through self-swabbing during unannounced home visits (14)	______ Collecting vaginal fluid samples through self-swabbing during unannounced home visits (14)

Q23.5 Lastly, we are interested in your opinions about how ethical these various approaches and technologies are. Please choose items and group them based on whether you think they are ethical, potentially unethical, or definitely unethical for detecting and measuring adherence

in ARV-based vaginal ring trials for women. You can select as many items from the list as you like for each category.

ETHICAL-- there is nothing unethical about doing this	POTENTIALLY UNETHICAL -- under some circumstances it could be unethical to do this	DEFINITELY UNETHICAL -- it would never be ethical to do this.
______ Magnetic tag in ring and hand-held meter during unannounced home visits (1)	______ Magnetic tag in ring and hand-held meter during unannounced home visits (1)	______ Magnetic tag in ring and hand-held meter during unannounced home visits (1)
______ Breath test for chemical in ring during unannounced home visits (4)	______ Breath test for chemical in ring during unannounced home visits (4)	______ Breath test for chemical in ring during unannounced home visits (4)
______ Measurement of residual drug in returned ring (2)	______ Measurement of residual drug in returned ring (2)	______ Measurement of residual drug in returned ring (2)
______ Sensor to measure exposure to temperature, air, pH, or vaginal pressure in returned ring (3)	______ Sensor to measure exposure to temperature, air, pH, or vaginal pressure in returned ring (3)	______ Sensor to measure exposure to temperature, air, pH, or vaginal pressure in returned ring (3)
______ Diffusion of vaginal substance into the returned ring (5)	______ Diffusion of vaginal substance into the returned ring (5)	______ Diffusion of vaginal substance into the returned ring (5)
______ Diffusion of safe, inactive substance out of returned ring (6)	______ Diffusion of safe, inactive substance out of returned ring (6)	______ Diffusion of safe, inactive substance out of returned ring (6)
______ Collecting hair samples during clinic visits (7)	______ Collecting hair samples during clinic visits (7)	______ Collecting hair samples during clinic visits (7)
______ Collecting blood samples during unannounced home visits (8)	______ Collecting blood samples during unannounced home visits (8)	______ Collecting blood samples during unannounced home visits (8)
______ Collecting vaginal fluid samples through self-swabbing during unannounced home visits (9)	______ Collecting vaginal fluid samples through self-swabbing during unannounced home visits (9)	______ Collecting vaginal fluid samples through self-swabbing during unannounced home visits (9)

Q23.6 Are there any other methods for assessing vaginal ring adherence that we've missed that you think should be considered? If yes, please describe.

Q24.1 Providing participants feedback on adherence measures Not all biometric measures would allow for real-time feedback to trial participants about their adherence but some would. We would like to hear your opinion on giving feedback to trial participants about their adherence, based on the kinds of biometric measures we have described in this survey.   Do

you think participants should be told what their adherence levels are during a trial, based on biometric measures?

- ❍ Yes (1)
- ❍ No (2)
- ❍ Don't know (3)

Q24.2 How often in the course of a year do you think adherence counselors should meet with participants to give them feedback on their adherence levels, based on biometric measures?

- ❍ Once per year (2)
- ❍ 2-4 times per year (3)
- ❍ At every follow-up visit (6)
- ❍ Never, I don't think counselors should give participants feedback on adherence based on biometric measures (1)
- ❍ Don't know (5)

Q24.3 How acceptable to trial staff would it be to them to give women feedback on their adherence based on biometric measures?

- ❍ Very acceptable (1)
- ❍ Somewhat acceptable (2)
- ❍ Not acceptable (3)
- ❍ Don't know (4)

Q24.4 How acceptable to trial participants would it be for them to receive feedback on their adherence based on biometric measures?

- ❍ Very acceptable (1)
- ❍ Somewhat acceptable (2)
- ❍ Not acceptable (3)
- ❍ Don't know (4)

Q24.5 How helpful to trial participants would it be for them to receive feedback on their adherence from study staff/counselors?

- ❍ Very helpful (1)
- ❍ Somewhat helpful (2)
- ❍ Not helpful (3)
- ❍ Don't know (4)

Q24.6 We are also interested in what you think would be some of the advantages and disadvantages of providing participants with feedback on their adherence as detected with the above approaches and biometric technologies.
ADVANTAGES of providing participants with feedback on their adherence Do you think the following are advantages that would result from giving participants feedback on their adherence?

	Yes (1)	No (2)	Don't know (3)
It would be an incentive for participants to maintain or improve their product adherence (1)	❍	❍	❍
Staff would be able to encourage non-users to use/adhere to the study product (2)	❍	❍	❍
It would provide an opportunity for more open dialogue and improved rapport between study participants and staff (3)	❍	❍	❍
Overall trial results would be more valid; would better assess efficacy of study product (4)	❍	❍	❍
Study funding and resources could be saved in the long run if results were more accurate (5)	❍	❍	❍
Participants might appreciate having an objective measure of their adherence (6)	❍	❍	❍
It’s likely more adherent women than non-adherent women would enroll in future trials if they were made aware of this practice (7)	❍	❍	❍
Other. Please specify: (8)	❍	❍	❍

Q24.7 DISADVANTAGES of providing participants with feedback on their adherence Do you think the following are disadvantages that would result from giving participants feedback on their drug levels or other adherence markers?

	Yes (1)	No (2)	Don't know (3)
It would alienate or offend trial participants (1)	❍	❍	❍
It would weaken the rapport between study staff and participants (2)	❍	❍	❍
Participants who didn't use or adhere well would be more likely to drop out of the trial once they were given feedback (3)	❍	❍	❍
Fewer people would participate in future trials once people became aware of this practice (4)	❍	❍	❍
It would require additional scarce resources and time for staff to meet with participants (5)	❍	❍	❍
Participants would just find more ways to outsmart the measures of adherence (6)	❍	❍	❍
Participants would not acknowledge or agree with the objective measures (7)	❍	❍	❍
Other. Please specify: (8)	❍	❍	❍

Q24.8 Should participants be given incentives for "good adherence" based on such measures? If yes, what kind of incentives? (e.g., money, gifts, certificates, etc.)
- ❍ Yes (1) ____________________
- ❍ No (2)
- ❍ Don't know (3)

Q24.9 If you have any additional comments on providing participants with feedback on their drug levels or other adherence markers, please note them here:

Q25.1 Outsmarting adherence measures In this survey we have described a number of different ways that adherence could be measured during vaginal ring HIV prevention trials. This included biometric and psychometric measures. In general, how likely do you think it is that vaginal ring trial participants would try to trick or outsmart adherence measures?
- ❍ Many or most participants would try to outsmart such measures (1)
- ❍ Some but not most participants would try to outsmart such measures (2)
- ❍ A few participants would try to outsmart such measures (3)
- ❍ Don't know (4)

Q25.2 We would like to know which adherence measurement approaches you think trial participants would be most likely to trick or outsmart and which they would be least likely to trick or outsmart. Please choose items from the list and drag them to the appropriate box. You can select as many items as you wish from the list for each category.

Adherence measures that trial participants would be MOST LIKELY to trick or outsmart	Adherence measures that trial participants would be LEAST LIKELY to trick or outsmart
______ Magnetic tag in ring and hand-held meter during unannounced home visits (1)	______ Magnetic tag in ring and hand-held meter during unannounced home visits (1)
______ Breath test for chemical in ring during unannounced home visits (2)	______ Breath test for chemical in ring during unannounced home visits (2)
______ Measurement of residual drug in returned ring (3)	______ Measurement of residual drug in returned ring (3)
______ Sensor to measure exposure to temperature, air, pH, or vaginal pressure in returned ring (4)	______ Sensor to measure exposure to temperature, air, pH, or vaginal pressure in returned ring (4)
______ Diffusion of vaginal substance into returned ring (5)	______ Diffusion of vaginal substance into returned ring (5)
______ Diffusion of safe, inactive substance out of returned ring (6)	______ Diffusion of safe, inactive substance out of returned ring (6)
______ Collecting hair samples during clinic visits (7)	______ Collecting hair samples during clinic visits (7)
______ Collecting blood samples during unannounced home visits (8)	______ Collecting blood samples during unannounced home visits (8)
______ Collecting vaginal fluid sample by self-swab during unannounced home visits (9)	______ Collecting vaginal fluid sample by self-swab during unannounced home visits (9)

______ Psychometric scale measures (10)	______ Psychometric scale measures (10)

Q27.1 Finally, we'd like to ask three general demographic questions. Your answers to these questions will help us understand the diversity of the people taking this survey. In which geographic region do you currently reside?

- North America (1)
- South America, Central America, and the Caribbean (2)
- Sub-Saharan Africa (3)
- Middle East and North Africa (4)
- South Asia (5)
- Central Asia (6)
- Southeast Asia (7)
- Europe or Australia (8)
- Other; please describe: (9) ____________________

Q27.2 What is the highest degree or level of school you have completed?

- No college (1)
- Some college or technical certificate (2)
- Undergraduate college degree (3)
- Master's level degree (4)
- Doctoral level degree (5)

Q27.3 What is your gender?

- Female (1)
- Male (2)
- Transgender (3)

Q28.1 Confidentiality in survey   Were any of your responses in this survey influenced by concerns over confidentiality?

- Yes (1)
- No (4)

Q29.1 If you have any additional comments you would like to share with us, please enter them here.

Q29.2 Thank you very much for your participation! We appreciate your time and input. To submit your survey, please click the forward arrow at the bottom of the page. Thank you.
